# Stochastic Properties of Fractional Generalized Cumulative Residual Entropy and Its Extensions

**DOI:** 10.3390/e24081041

**Published:** 2022-07-28

**Authors:** Ghadah Alomani, Mohamed Kayid

**Affiliations:** 1 Department of Mathematical Sciences, College of Science, Princess Nourah bint Abdulrahman University, P.O. Box 84428, Riyadh 11671, Saudi Arabia; gaalomani@pnu.edu.sa; 2 Department of Statistics and Operations Research, College of Science, King Saud University, P.O. Box 2455, Riyadh 11451, Saudi Arabia

**Keywords:** FGCRE, generalized cumulative residual entropy, mean residual lifetime, stochastic orders

## Abstract

The fractional generalized cumulative residual entropy (FGCRE) has been introduced recently as a novel uncertainty measure which can be compared with the fractional Shannon entropy. Various properties of the FGCRE have been studied in the literature. In this paper, further results for this measure are obtained. The results include new representations of the FGCRE and a derivation of some bounds for it. We conduct a number of stochastic comparisons using this measure and detect the connections it has with some well-known stochastic orders and other reliability measures. We also show that the FGCRE is the Bayesian risk of a mean residual lifetime (MRL) under a suitable prior distribution function. A normalized version of the FGCRE is considered and its properties and connections with the Lorenz curve ordering are studied. The dynamic version of the measure is considered in the context of the residual lifetime and appropriate aging paths.

## 1. Introduction

The classical Shannon entropy (see Shannon [1]) associated with a random variable (RV) *X* has a crucial role in many branches of science to measure the uncertainty contained in X. Throughout the paper, *X* denotes a non-negative RV with an absolutely continuous cumulative distribution function (CDF) with corresponding probability density function (PDF) f. The Shannon differential entropy is

(1) $$H\left(X\right)=-\int_{0}^{\infty }f\left(x\right)\log f\left(x\right)dx.$$

Possible alternative measures of information have been introduced in the literature.

The cumulative residual entropy (CRE) initiated by Rao et al. [2] as a counterpart to (1), obtained by substituting the survival function (SF) S≡1−F in place of the PDF f, as

(2) $$E\left(X\right)=-\int_{0}^{+\infty }S\left(x\right)\log S\left(x\right)dx=\int_{0}^{+\infty }S\left(x\right)\Omega \left(x\right)dx,$$

where

(3) $$\Omega \left(x\right)=-\log S\left(x\right)=\int_{0}^{x}\lambda \left(u\right)du,\;x>0,$$

is the cumulative the hazard rate (HR) function and $\lambda \left(t\right)=\frac{f(t)}{S(t)},\;t>0,$ is the HR function. Dynamic versions of the CRE were considered in Asadi and Zohrevand [3] and also in Navarro et al. [4] where the CRE of the residual lifetime $X_{t}=\left(X-t|X>t\right)$ was measured as

$$E\left(t\right)=E\left(X;t\right)=-\int_{t}^{\infty }\frac{S(x)}{S(t)}\log\frac{S(x)}{S(t)}dx,\;t>0.$$

For related results, one can see Baratpour [5], Baratpour and Habibi Rad [6] and also Toomaj et al. [7] and the references therein. In a recent work by Di Crescenzo et al. [8], the CRE measure was extended to FGCRE as

(4) $$E_{\alpha }\left(X\right)=c\left(\alpha \right)\int_{0}^{\infty }S\left(x\right){\left[-\log S\left(x\right)\right]}^{\alpha }dx,$$

where $c\left(\alpha \right)=\frac{1}{\Gamma (\alpha +1)},\;\alpha \geq 0$. The notation c(α) is used across the paper. Note that $c\left(n\right)=\frac{1}{n!}.$ The properties of fractional cumulative entropy, such as its alteration under linear transformations, its bounds, its connection to stochastic orders along with its empirical estimation, and various relations to other functions have been argued and discussed by Xiong et al. [9]. We note that, as pointed out by [8], if α is a positive integer, say, α=n∈N, then $E_{n}\left(X\right)$ is identical to the generalized cumulative residual entropy (GCRE) introduced by Psarrakos and Navarro [10]. It is noticeable that $E_{n}\left(X\right)$ is considered a dispersion measure. The measure is also connected to the relevance transformation and interepoch intervals of a nonhomogeneous Poisson process (see, e.g., Toomaj and Di Crescenzo [11]). This paper aims to continue this line of research. In this context, we present new findings on the FGRCE and its dynamic version. The FGCRE is in particular a suitable quantity to be applied in the proportional HR model.

The subsequent materials of this article are organized in the following order. In Section 2, we first give an overview of the concept of generalized cumulative residual entropy and present a similar representation for fractional generalized residual cumulative entropy. We then give some expressions for the FGCRE, one of which is related to the MRL function. We also consider the connection of the FGCRE with the excess wealth order and the Bayesian risk of the FGCRE. A normalized version of the FGCRE is given and its connection with the Lorenz curve order is studied. Section 3 examines some bounds and stochastic ordering properties of FGCRE. In Section 4, properties of the dynamic FGCRE are discussed.

The reader can be referred to [12] for the definitions of stochastic orders $\leq_{st},\leq_{hr},\leq_{lr},$$\leq_{ew}$ and $\leq_{Lorenz}$ and for the definitions of (increasing) decreasing MRL (IMRL(DMRL)), (decreasing) increasing failure rate (DFR (IFR)) and new better (worse) than used in expectation (NBUE (NWUE)) classes.

## 2. Basic Properties

As mentioned earlier, the FGCRE in (4) reduces to the GCRE when α=n∈N. In this case,

(5) $$E_{n}\left(X\right)=c\left(n\right)\int_{0}^{\infty }S\left(x\right){\left[\Omega \left(x\right)\right]}^{n}\;dx=c\left(n\right)\int_{0}^{\infty }S\left(x\right){\left[-\log S\left(x\right)\right]}^{n}\;dx$$

for all n=0,1,…. As pointed out by Psarrakos and Navarro [10], the GCRE fulfills the following property:

(6) $$E_{n}\left(X\right)=\mu_{n+1}-\mu_{n},\;n\geq 0,$$

where $\mu_{n}=E\left[X_{n+1}\right]$ and $X_{n}$ denotes the epoch times of a Poisson process which is nonhomogeneous having intensity function λ(x). Note that $X_{1}$ and *X* are equally distributed. Signifying by $S_{n+1}\left(x\right)$ the SF of $X_{n+1},$n∈{0,1,2,…}, one has (see Baxter [13])

(7) $$S_{n+1}\left(x\right)=S\left(x\right)\;\sum_{k=0}^{n}\frac{\Omega^{k}\left(x\right)}{k!},\;x\geq 0,$$

and the PDF of $X_{n+1}$ is

(8) $$f_{n+1}\left(x\right)=c\left(n\right)f\left(x\right)\Omega^{n}\left(x\right),\;x\geq 0.$$

In the following, we show that the same results can be obtained for the FGCRE. It is worth noting that our results are extensions of the results obtained using the GCRE. To this end, we define the RV $X_{\alpha +1}$ with the PDF as

(9) $$f_{\alpha +1}\left(x\right)=c\left(\alpha \right){\left[\Omega \left(x\right)\right]}^{\alpha }f\left(x\right),\;x\geq 0,$$

for all α>0 where Ω(x) is defined in (3). Denoting by $S_{\alpha +1}\left(x\right)$ the SF of $X_{\alpha +1},$ it can be represented as $S_{\alpha +1}\left(x\right)=B_{\alpha +1}\left(S\left(x\right)\right),\;x\geq 0,$ where

$$B_{\alpha }\left(t\right)=c\left(\alpha \right)\int_{0}^{t}{\left(-\log u\right)}^{\alpha }du,\;t\in \left(0,1\right),$$

is increasing in *t* for all α≥0. If α is an integer, say, α∈{0,1,2,…}, then (9) reduces to (8). Notice that from (4), the FGCRE can be rewritten as

(10) $$E_{\alpha }\left(X\right)=E\left(\frac{1}{\lambda (X_{\alpha +1})}\right),\;x>0,$$

for all α≥0. From (9), the ratio

$$\frac{f_{\alpha_{2}}\left(x\right)}{f_{\alpha_{1}}\left(x\right)}=\frac{c(\alpha_{2})}{c(\alpha_{1})}{\left(\Omega (x)\right)}^{\alpha_{2}-\alpha_{1}},\;x>0,$$

is increasing in *t* and, therefore, $X_{\alpha_{1}}\leq_{lr}X_{\alpha_{2}}$ for any $0<\alpha_{1}\leq \alpha_{2}.$ In particular, this implies that $X_{\alpha_{1}}\leq_{st}X_{\alpha_{2}}$. That is, $S_{\alpha_{1}}\left(x\right)\leq S_{\alpha_{2}}\left(x\right)$ for all $0<\alpha_{1}\leq \alpha_{2}$. Hence, if *X* is IFR (DFR), then, from (10) and Equation (1.A.7) in [12], we have

(11) $$E_{\alpha_{1}}\left(X\right)\leq \left(\geq \right)E_{\alpha_{2}}\left(X\right),$$

for all $\alpha_{1}\leq \alpha_{2}$. In Table 1, we give FGCREs for a number of distributions.

Now, we obtain an analogue representation for the FGCRE which is a generalization of relation (6) with FGCRE in place of GCRE.

**Proposition** **1.** *Let X have FGCRE $E_{\alpha }\left(X\right).$ Then, for all α≥0,*

(12) $$E_{\alpha }\left(X\right)=E\left[X_{\alpha +1}\right]-E\left[X_{\alpha }\right].$$

**Proof.**  Recalling (4) and integrating by parts, we obtain

$$\begin{array}{ccc} E_{\alpha }\left(X\right) & = & c\left(\alpha \right)\left\{\int_{0}^{\infty }x{\left[\Omega \left(x\right)\right]}^{\alpha }f\left(x\right)dx-\alpha \int_{0}^{\infty }x{\left[\Omega \left(x\right)\right]}^{\alpha -1}f\left(x\right)dx\right\}\\ & = & \int_{0}^{\infty }xf_{\alpha +1}\left(x\right)dx-\frac{\alpha c(\alpha +1)}{c(\alpha )}\int_{0}^{\infty }xf_{\alpha }\left(x\right)dx\\ & = & E\left[X_{\alpha +1}\right]-E\left[X_{\alpha }\right], \end{array}$$

where the last equality is obtained by recalling (9) and using c(α)=αc(α+1). □

**Figure 1:**
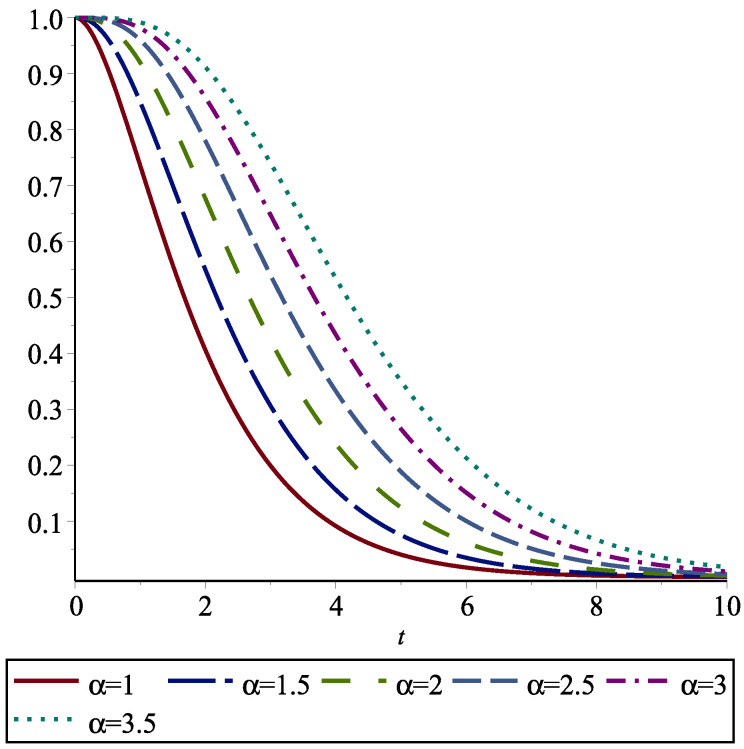
$S_{\alpha }\left(x\right)$ for an exponential distribution for α=1,1.5,2,2.5,3,3.5. The area under $S_{1}\left(x\right)=S\left(x\right)$ is $\bar{E}\left(X\right)$ and the areas among them give the amounts of the FGCRE $E_{\alpha }\left(X\right)$ for α=1,1.5,2,2.5,3,3.5.

Note that $E_{\alpha }\left(X\right)$ is the areas surrounded between $S_{\alpha +1}$ and $S_{\alpha }$ for all α≥0. In particular, $S_{0}=E\left(X\right)$ is the area under $S_{1}=S$. In Figure 1, we depict these areas for the exponential distribution and various values of α.

**Theorem** **1.** **(i)** *If, for some* p>1/α, $E(X^{p})<\infty ,$*then* $E_{\alpha }\left(X\right)<\infty$*for all* 0<α≤1.

**(ii)** *If, for some* p>α, $E(X^{p})<\infty ,$*then* $E_{\alpha }\left(X\right)<\infty$*for all* α≥1.

**Proof.**  (i) It is not difficult to see whether for each 0≤α≤1, and 0≤β≤1, one can obtain

(13) $$x{\left(-\log x\right)}^{\alpha }\leq {\left(\frac{\alpha e^{-1}}{1-\beta }\right)}^{\alpha }x^{\beta },\;0\leq x\leq 1,$$

By taking β=α for 0≤α≤1, we obtain

$$x{\left(-\log x\right)}^{\alpha }\leq {\left(\frac{\alpha e^{-1}}{1-\alpha }\right)}^{\alpha }x^{\alpha },\;0\leq x\leq 1.$$

Thus, one concludes

$$\begin{array}{ccc} E_{\alpha }\left(X\right) & \leq & {\left(\frac{\alpha e^{-1}}{1-\alpha }\right)}^{\alpha }\int_{0}^{\infty }S^{\alpha }\left(x\right)dx={\left(\frac{\alpha e^{-1}}{1-\alpha }\right)}^{\alpha }\left[\int_{0}^{1}S^{\alpha }\left(x\right)dx+\int_{1}^{\infty }S^{\alpha }\left(x\right)dx\right]\\ & \leq & {\left(\frac{\alpha e^{-1}}{1-\alpha }\right)}^{\alpha }\left[1+\int_{1}^{\infty }S^{\alpha }\left(x\right)dx\right]\leq {\left(\frac{\alpha e^{-1}}{1-\alpha }\right)}^{\alpha }\left[1+\int_{1}^{\infty }{\left[\frac{E(X^{p})}{x^{p}}\right]}^{\alpha }dx\right]\\ & = & {\left(\frac{\alpha e^{-1}}{1-\alpha }\right)}^{\alpha }\left[1+{\left[E\left(X^{p}\right)\right]}^{\alpha }\int_{1}^{\infty }\frac{1}{x^{\alpha p}}dx\right], \end{array}$$

where the third inequality is obtained by virtue of the Markov inequality. The last expression is finite if $p>\frac{1}{\alpha }$ and this completes the proof. In the case when α≥1, the results apply to β=1/α. □

Note that $E_{\alpha }\left(X\right)=E_{\alpha }\left(Y\right),\;\alpha \geq 0,$ does not guarantee equality in the distributions of *X* and *Y*, but the converse holds. If Y=i(X), where i(·) is strictly increasing and differentiable, then

(14) $$E_{\alpha }\left(Y\right)=c\left(\alpha \right)\int_{0}^{\infty }i^{\prime }\left(u\right)S\left(u\right){\left[-\log S\left(u\right)\right]}^{\alpha }du,$$

for all α≥0. Below, the connection between the FGCRE and the cumulative HR function of *X* given by (3) is realized.

**Theorem** **2.** *Let X fulfill* $E_{\alpha }\left(X\right)<+\infty$*for all* α≥0*. Then,*

(15) $$E_{\alpha }\left(X\right)=E\left[\Omega_{\alpha }^{\left(2\right)}\left(X\right)\right],$$

*where*

(16) $$\Omega_{\alpha }^{\left(2\right)}\left(x\right)=c\left(\alpha \right)\int_{0}^{x}\Omega^{\alpha }\left(t\right)dt,\;x\geq 0.$$

**Proof.**  From (4) and also by applying Fubini’s theorem,

$$E_{\alpha }\left(X\right)=c\left(\alpha \right)\int_{0}^{\infty }\left[\int_{t}^{\infty }f\left(x\right)dx\right]\Omega^{\alpha }\left(t\right)dt=c\left(\alpha \right)\int_{0}^{\infty }f\left(x\right)\left[\int_{0}^{x}\Omega^{\alpha }\left(t\right)dt\right]dx,$$

which immediately validates (15) by using (16). □

We note that $\Omega_{\alpha }^{\left(2\right)}\left(x\right)$ in (16) is increasing and convex in x. This immediately generates the following property.

**Theorem** **3.** *Let X have a finite mean μ. Then,*

$$E_{\alpha }\left(X\right)\geq \Omega_{\alpha }^{\left(2\right)}\left(\mu \right),$$

*for all α≥0.*

Another useful application of Theorem 2 is given here.

**Theorem** **4.** *If X and Y are non-negative RVs in the way $X\leq_{icx}Y$, it holds that*

$$\Omega_{\alpha }^{\left(2\right)}\left(X\right)\leq_{icx}\Omega_{\alpha }^{\left(2\right)}\left(Y\right),\;\alpha \geq 0,$$

*where the function $\Omega_{\alpha }^{\left(2\right)}\left(\cdot \right)$ is given in (16). In particular, $X\leq_{icx}Y$ implies*

$$E_{\alpha }\left(X\right)\leq E_{\alpha }\left(Y\right).$$

**Proof.**  Since $\Omega_{\alpha }^{\left(2\right)}\left(\cdot \right)$ is a convex function and also since it is an increasing function for all α≥0, thus (see Theorem 4.A.8 in [12]), $\Omega_{\alpha }^{\left(2\right)}\left(X\right)\leq_{icx}\Omega_{\alpha }^{\left(2\right)}\left(Y\right),\;\alpha \geq 0$. Now, using relation 4.A.2 in [12], we derive $E_{\alpha }\left(X\right)\leq E_{\alpha }\left(Y\right)$. □

Clearly, $\Omega_{\alpha }^{\left(2\right)}\left(\cdot \right)$ is increasing and also convex and $\Omega_{\alpha }^{\left(2\right)}\left(0\right)=0.$ Hence, for the RVs *X* and *Y* satisfying $X\leq_{hr}Y,$ we obtain that

(17) $$\frac{E_{\alpha }\left(X\right)}{E(X)}\leq \frac{E_{\alpha }\left(Y\right)}{E(Y)},$$

for all α≥0. This relation is immediately obtained from Theorem 2 and Shaked and Shantikumar [12] (see page 24). It is worth pointing out that Equation (17) leads us to define the normalized FGCRE by

(18) $${\mathrm{NE}}_{\alpha }\left(X\right)=\frac{E_{\alpha }\left(X\right)}{E(X)}.$$

Under the condition $X\leq_{hr}Y,$ Equation (17) can be rewritten as ${\mathrm{NE}}_{\alpha }\left(X\right)\leq {\mathrm{NE}}_{\alpha }\left(Y\right)$ for α≥0. Moreover, if *X* is a non-negative RV having IFR (DFR) property, from relation (11), one can conclude that

$$E_{0}\left(X\right)\geq \left(\leq \right)E_{\alpha }\left(X\right),\;\mathrm{for}\;\mathrm{all}\;\alpha \geq 0.$$

From this, we derive that ${\mathrm{NE}}_{\alpha }\left(X\right)\leq \left(\geq \right)1,\;\alpha \geq 0.$ For α=1, the normalized cumulative residual entropy ${\mathrm{NE}}_{1}\left(X\right)$ is generated (see Rao [2]). This is an analogue for the coefficient of variation of an RV. In Table 2, we give the normalized FGCREs for some distributions.

To continue our results, consider the following observation.

**Theorem** **5.** *Let $E_{\alpha }\left(X\right)<+\infty$ for all α≥0. Then*

(19) $${\mathrm{NE}}_{\alpha }\left(X\right)=\int_{0}^{1}\left[p-L_{X}\left(p\right)\right]g_{\alpha }\left(p\right)dp,$$

*where*

$$g_{\alpha }\left(p\right)=\frac{{\left[-\log\left(1-p\right)\right]}^{\alpha -2}}{\Gamma (\alpha )(1-p)}\left[-\log\left(1-p\right)-\left(\alpha -1\right)\right],\;0\leq p\leq 1.$$

**Proof.**  Recalling Proposition 1 and the change of z=F(x), we have

(20) $${\mathrm{NE}}_{\alpha }\left(X\right)=\frac{1}{\mu }\int_{0}^{1}\left[F^{-1}\left(z\right)-\mu \right]G_{\alpha }\left(z\right)dz,$$

where

$$G_{\alpha }\left(z\right)=\alpha {\left[-\log\left(1-z\right)\right]}^{\alpha }-\frac{{\left[-\log\left(1-z\right)\right]}^{\alpha -1}}{\Gamma (\alpha )}+1,\;0\leq z\leq 1,$$

for all α>0. In (20), let $u=G_{\alpha }\left(z\right),\;\alpha \geq 0,$ and $dv=[F^{-1}\left(z\right)-\mu ]dz.$ Then $du=g_{\alpha }\left(z\right)dz$ and $v=\int_{0}^{p}\left[F^{-1}\left(z\right)-\mu \right]dz.$ Integrating by parts gives

$${\mathrm{NE}}_{\alpha }\left(X\right)=\int_{0}^{1}g_{\alpha }\left(p\right)\int_{0}^{p}\left[1-\frac{F^{-1}\left(z\right)}{\mu }\right]dp,$$

and this gives the proof. □

When α=1, the De Vergottini index of inequality of an income distribution *X* is reached, given by ${\mathrm{NE}}_{1}\left(X\right)=E_{1}\left(X\right)/E(X)$ (see Rao et al. [2] for more details). The index (19) belongs to the class of linear measures of income inequality defined by Mehran [14]. It can be obtained by weighting the Lorenz differences $p-L_{X}\left(p\right)$ together with the income distribution.

**Theorem** **6.** *Let $X_{1}$ and $X_{2}$ be non-negative RVs with survival functions $S_{1}\left(x\right)$ and $S_{2}\left(x\right),$ respectively. If $X_{1}\leq_{Lorenz}X_{2},$ then ${\mathrm{NE}}_{\alpha }\left(X_{1}\right)\leq {\mathrm{NE}}_{\alpha }\left(X_{2}\right)$ for all 0≤α≤1.*

**Proof.**  Assumption $X_{1}\leq_{Lorenz}X_{2}$ implies that $L_{X}\left(p\right)\geq L_{Y}\left(p\right),\;p\in \left[0,1\right],$ due to Theorem 3.A.10 in [12]. From relation (19), we obtain

$$\left[p-L_{X}\left(p\right)\right]g_{\alpha }\left(p\right)\leq \left[p-L_{Y}\left(p\right)\right]g_{\alpha }\left(p\right),\;p\in \left[0,1\right],$$

where the inequality is obtained by noting that $g_{\alpha }\left(p\right),\;0\leq p\leq 1,$ is a non-negative function for all 0≤α≤1. The result is obtained by reversing. □

### The Bayes Risk of MRL

The PDF of $X_{t}$ is given by $f_{t}\left(x\right)=f\left(x\right)/S\left(t\right)$ for x>t. Denote by m(t) the MRL function of *X*. In the decision theoretic framework, the MRL function is the optimal prediction of [X−t|X>t], under the conditional quadratic loss function $L\left(d,X|t\right)=[{\left(X-t-d\right)}^{2}|X>t],$ as the mean of the PDF $f_{t}\left(x\right).$ In other words, we have

$$d^{★}\left(t\right)=\arg\min_{d}E_{X>t}\left[L\left(d,X|t\right)\right]=m\left(t\right),\;t>0,$$

for all α>0. The function m(t) is a local risk measure, given the value the threshold *t* takes. Its global risk of the MRL function of *X* is the Bayes risk

(21) $$E\left(m\right)=E_{\pi }\left[m\left(X\right)\right],$$

where $E_{\pi }$ denotes the average based on the prior PDF for the threshold *t* (see Ardakani et al. [15] and Asadi et al. [16] for more details). The following theorem provides expressions for E(m) under different priors.

**Theorem** **7.** *Let X have the MRL function m, and let $\pi \left(t\right)=f_{\alpha }\left(t\right),\;t\geq 0.$ Then, the Bayes risk of m(t) is given by the FGCRE functional of the baseline CDF, i.e.,*

(22) $$E\left(m\right)=E_{\alpha }\left(X\right).$$

**Proof.**  By substituting $\pi \left(t\right)=f_{\alpha }\left(t\right)=c\left(\alpha -1\right){\left[\Omega \left(t\right)\right]}^{\alpha -1}f\left(t\right),\;t\geq 0,$ for all α>0, we have

$$\begin{array}{ccc} E(m) & = & \int_{0}^{\infty }m\left(t\right)\pi \left(t\right)dt=\int_{0}^{\infty }m\left(t\right)f_{\alpha }\left(t\right)dt\\ & = & \int_{0}^{\infty }\left(\frac{\int_{t}^{\infty }S\left(x\right)dx}{S(t)}\right)f_{\alpha }\left(t\right)dt\\ & = & \int_{0}^{\infty }S\left(x\right)\left(\int_{0}^{x}\frac{f_{\alpha }\left(t\right)}{S(t)}dt\right)dx\\ & = & c\left(\alpha \right)\int_{0}^{\infty }S\left(x\right){\left[-\log S\left(x\right)\right]}^{\alpha }dx. \end{array}$$

The second equality follows by observing that

$$\int_{0}^{x}\frac{f_{\alpha }\left(t\right)}{S(t)}dt=c\left(\alpha -1\right)\int_{0}^{x}{\left[\Omega \left(t\right)\right]}^{\alpha -1}f\left(t\right)dt=c\left(\alpha \right){\left[\Omega \left(t\right)\right]}^{\alpha },\;t\geq 0,$$

and the proof is completed. □

From Theorem 7, it is obvious that

(23) $$E_{\alpha }\left(X\right)=E\left[m\left(X_{\alpha }\right)\right],$$

for all α≥0. We point out that the representation in (23) is very useful since in many statistical models one may gather information about the behaviour of MRL. The following example illustrates a well-known situation in this context.

**Example** **1.** *Let us suppose m(x)=cx+d, x≥0, with c>−1, c≠0 and d>0. Oakes and Dasu [17] observed that the corresponding SF is*

$$S\left(x\right)={\left(\frac{d}{cx+d}\right)}^{\frac{1}{c}+1},\;x\geq 0,\;c>-1,\;d>0.$$

*It is a well-known property for the generalized Pareto distribution (GPD) as a fundamental aspect of this family of distributions. The exponential distribution is reached whenever c→0, the Pareto distribution is resulted for c>0, and the power distribution is achieved for −1<c<0. Hence, from (23), the FGCRE of the GPD distribution is derived as*

$$E_{\alpha }\left(X\right)=cE\left[X_{\alpha }\right]+d=d{\left(c+1\right)}^{\alpha },$$

*where the identity $E\left[X_{\alpha }\right]=\frac{d}{c}\left[{\left(c+1\right)}^{\alpha }-1\right],$ for all α≥0, has been applied.*

The Bayes risk of m(t) under the prior $\pi \left(t\right)=f_{\alpha }\left(t\right)$ is given by $E_{\alpha }\left(X\right)\leq \left(\geq \right)\mu$ for all α≥0.

## 3. Bounds and Stochastic Ordering

In this section, we aim to derive several results on bounds for the FGCRE and provide results based on stochastic comparisons.

### 3.1. Some Bounds

It is well known that the cumulative residual entropy of the sum of two non-negative independent RVs is greater than the maximum of their original entropies (see, for example, Rao et al. [2]). By a similar approach, we can verify that the same result also holds true for the FGCRE. We omit the proof.

**Theorem** **8.** *If $X_{1}$ and $X_{2}$ are non-negative independent RVs, then*

$$E_{\alpha }\left(X_{1}+X_{2}\right)\geq \max\left\{E_{\alpha }\left(X_{1}\right),E_{\alpha }\left(X_{2}\right)\right\},$$

*for all α≥0.*

The following theorem establishes a bound for the FGCRE in terms of the cumulative residual entropy (2).

**Theorem** **9.** *Let X have a finite mean μ and finite E(X). Then*

(24) $$E_{\alpha }\left(X\right)\left\{\begin{array}{c} \leq \frac{c\left(\alpha \right){\left[E\left(X\right)\right]}^{\alpha }}{\mu^{\alpha -1}},\;\mathrm{if}\;0\leq \alpha \leq 1\\ \geq \frac{c\left(\alpha \right){\left[E\left(X\right)\right]}^{\alpha }}{\mu^{\alpha -1}},\;\mathrm{if}\;\alpha \geq 1 \end{array}.\right.$$

**Proof.**  Let $X_{e}$ follow the equilibrium distribution with PDF $f_{e}\left(x\right)=S\left(x\right)/\mu ,\;x>0.$ The FGCRE can be rewritten as

$$E_{\alpha }\left(X\right)=\mu E\left[\psi_{\alpha }\left(\Omega \left(X_{e}\right)\right)\right],$$

in which $\psi_{\alpha }\left(t\right)=c\left(\alpha \right)t^{\alpha },\;t>0,$ is a concave (convex) function for 0≤α≤1(α≥1). Therefore, Jensen’s inequality implies

$$\begin{array}{ccc} E_{\alpha }\left(X\right) & = & \mu E[\psi_{\alpha }\left(\Omega \left(X_{e}\right)\right)]\\ & \leq & \mu c\left(\alpha \right)\psi_{\alpha }\left(E[\Omega \left(X_{e}\right)]\right)\\ & = & \mu c\left(\alpha \right){\left(\frac{1}{\mu }\int_{0}^{+\infty }S\left(x\right)\Omega \left(x\right)dx\right)}^{\alpha }, \end{array}$$

and this provides the proof in the spirit of (2). If α≥1, the result is obtained analogously. □

In the setting of Theorem 9, the properties given below hold for the normalized FGCRE.

(25) $${\mathrm{NE}}_{\alpha }\left(X\right)\left\{\begin{array}{c} \leq c\left(\alpha \right){\left[\mathrm{NE}\left(X\right)\right]}^{\alpha },\;\mathrm{if}\;0\leq \alpha \leq 1\\ \geq c\left(\alpha \right){\left[\mathrm{NE}\left(X\right)\right]}^{\alpha },\;\mathrm{if}\;\alpha \geq 1 \end{array}.\right.$$

**Theorem** **10.** *If X has a finite* E(X),*then, for all* α≥0,

**(i)**  *$E_{\alpha }\left(X\right)\geq C_{\alpha }e^{H(X)}$ such that $C_{\alpha }=c\left(\alpha \right)e^{\int_{0}^{1}\log\left(x{\left(-\log\left(x\right)\right)}^{\alpha }\right)dx}$ and H(X) given by (1).*
**(ii)**  $E_{\alpha }\left(X\right)\geq c\left(\alpha \right)\int_{0}^{\infty }F^{\alpha }\left(x\right)S\left(x\right)dx.$

**Proof.**  Part (i) is easily obtained by applying the log-sum inequality (see, e.g., Rao et al. [2]). By using the identity logx≤x−1 for 0<x≤1, then part (ii) can be obtained. □

We end this subsection by providing two upper bounds for the FGCRE of X. The first one is based on standard deviation of X. The second one is based on the risk-adjusted premium introduced by Wang [18] which is defined by

(26) $$\pi_{q}\left(X\right)=\int_{0}^{\infty }S^{q}\left(x\right)dx,$$

for all 0<q≤1. The risk-adjusted premium is additive when the risk is divided into layers, which makes it very attractive for pricing insurance layers. For a detailed discussion, the reader is referred to Wang [18].

**Theorem** **11.** *Consider X with standard deviation σ(X) and FGCRE function $E_{\alpha }\left(X\right).$ Then*

**(i)**  *$E_{\alpha }\left(X\right)\leq \frac{\sqrt{\Gamma (2\alpha -1)}}{\Gamma (\alpha )}\sigma \left(X\right),$ for all α≥0.5.*
**(ii)**  $E_{\alpha }\left(X\right)\leq {\left(\frac{\alpha e^{-1}}{1-\beta }\right)}^{\alpha }\frac{\pi_{\beta }\left(X\right)}{\Gamma (\alpha +1)}$*where* β=α*for* 0≤α≤1*and* β=1/α*for* α≥1.

**Proof.**  (i) For all α≥0, by the Cauchy–Schwarz inequality, from (23) we obtain

$$\begin{array}{ccc} {\left[\int_{0}^{\infty }m\left(x\right)\Omega^{\alpha -1}\left(x\right)f\left(x\right)dx\right]}^{2} & = & {\left[\int_{0}^{\infty }m\left(x\right)\sqrt{f(x)}\sqrt{f(x)}\Omega^{\alpha -1}\left(x\right)dx\right]}^{2}\\ & \leq & \left(\int_{0}^{\infty }m^{2}\left(x\right)f\left(x\right)dx\right)\left(\int_{0}^{\infty }\Omega^{2\alpha -2}\left(x\right)f\left(x\right)dx\right). \end{array}$$

Applying Theorem 21 of Toomaj and Di Crescenzo [11], it holds that $E\left[m^{2}\left(X\right)\right]=\sigma^{2}\left(X\right).$ Further,

$$\int_{0}^{\infty }\Omega^{2\alpha -2}\left(x\right)f\left(x\right)dx=\Gamma \left(2\alpha -1\right),$$

which is positive for all α≥0.5. Therefore, the proof is then completed. Part (ii) is easily obtained from relation (13) by substituting β=α for 0≤α≤1 and β=1/α for α≥1. □

The standard deviation (SD) bound in Theorem 11 is decreasing in 1/2<α≤1 and increasing in α≥1, but it is applicable when α>1/2. However, the risk-adjustment (RA) bound is applicable for all α≥0. Therefore, this bound can be a useful alternative for the case of α<1/2. The following example illustrates these points.

**Example** **2.** *Consider X with SF $S\left(x\right)=e^{-x^{k}},\;x>0.$ Then,*

$$\pi_{\beta }\left(X\right)=\int_{0}^{\infty }S^{\beta }\left(x\right)dx=\int_{0}^{\infty }e^{-\beta x^{k}}dx=\frac{\Gamma (\frac{1}{k})}{k\beta^{\frac{1}{k}}},$$

*for all k,β>0. The variance and the FGCRE of the Weibull distribution as given in Table 1 are*

$$\sigma^{2}\left(X\right)=\Gamma \left(1+\frac{2}{k}\right)-{\left[\Gamma \left(1+\frac{1}{k}\right)\right]}^{2}\;\mathrm{and}\;E_{\alpha }\left(X\right)=\frac{\Gamma (\alpha +\frac{1}{k})}{k\Gamma (\alpha +1)},$$

*respectively. Therefore, part (i) of Theorem 11 gives*

$$E_{\alpha }\left(X\right)\leq c\left(\alpha -1\right)\sqrt{\Gamma \left(2\alpha -1\right)\left[\Gamma \left(1+\frac{2}{k}\right)-{\left[\Gamma \left(1+\frac{1}{k}\right)\right]}^{2}\right]},\;\alpha >1/2.$$

*Moreover, by taking β=α for all 0≤α≤1 and β=1/α for all α≥1, part (ii) of Theorem 11 gives*

$$E_{\alpha }\left(X\right)\leq \frac{\alpha^{\alpha -\frac{1}{k}}e^{-\alpha }\Gamma \left(\frac{1}{k}\right)}{k{\left(1-\alpha \right)}^{\alpha }\Gamma \left(\alpha +1\right)},\;0\leq \alpha \leq 1,\;\mathrm{and}\;E_{\alpha }\left(X\right)\leq \frac{\alpha^{2\alpha +\frac{1}{k}}e^{-\alpha }\Gamma \left(\frac{1}{k}\right)}{k{\left(\alpha -1\right)}^{\alpha }\Gamma \left(\alpha +1\right)},\;\alpha \geq 1.$$

**Figure 2:**
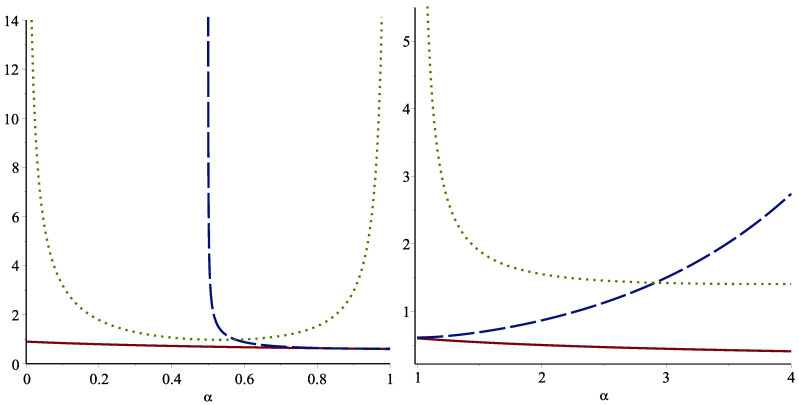
The SD (dashed line) and the RA (dotted line) bounds as well as the exact value of FGCRE (solid line) for the Weibull model with scale parameter k=2 when 0≤α≤1 (**left**) and α≥1 (**right**).

*The left panel of Figure 2 indicates the plots of the SD and the RA bounds given in Theorem 11 along with the plot of $E_{\alpha }\left(X\right)$ for 0≤α≤1, and the right panel is for α≥1. The standard deviation bound is not valid for 0≤α≤1/2. For 1/2≤α≤1, the standard deviation bound is outperformed.*

### 3.2. Stochastic Comparisons

In this subsection, ordering distributions according to the FGCRE is considered. We provide a counterexample to show that the usual stochastic ordering does not provide ordered distributions in accordance with their FGCREs.

**Example** **3.** *Let us consider two RVs $X_{1}$ and $X_{2}$ coming from the Weibull distribution with the survival functions $S_{1}\left(x\right)=e^{k_{1}-\frac{k_{1}}{x^{2}}}$ and $S_{2}\left(x\right)=e^{k_{2}-\frac{k_{2}}{x^{2}}}$ for all 0≤x≤1 and $k_{1},k_{2}>0.$ It is not hard to see that for $k_{1}\leq k_{2},$ we have $X_{1}\leq_{st}X_{2}.$ However, numerical computations illustrate that for some choices of $k_{1}$ and $k_{2}$ and for some choices of α, the condition $E_{\alpha }\left(X_{1}\right)\leq E_{\alpha }\left(X_{2}\right)$ is not fulfilled as shown in Table 3.*

Before stating our main results, let us consider the following lemma.

**Lemma** **1.** *If $X_{1}\leq_{st}X_{2},$ then $X_{1,\alpha }\leq_{st}X_{2,\alpha }$ for all α≥0.*

**Proof.**  The SF of $X_{i,\alpha },i=1,2,$ is $S_{i,\alpha }\left(x\right)=B_{\alpha }\left(S_{i}\left(x\right)\right),x>0.$ Since $X_{1}\leq_{st}X_{2},$ we have

$$S_{1,\alpha }\left(x\right)=B_{\alpha }\left(S_{1}\left(x\right)\right)\leq B_{\alpha }\left(S_{2}\left(x\right)\right)=S_{2,\alpha }\left(x\right),\;x>0,$$

in which the inequality follows since $B_{\alpha }\left(t\right)$ is increasing in t. Hence, the proof is completed. □

**Theorem** **12.** *Let* $X_{1}\leq_{st}X_{2}$*. Then, for all* α≥0:

**(i)**  *If $X_{1}\leq_{mrl}X_{2}$ and either $X_{1}$ or $X_{2}$ is IMRL, then $E_{\alpha }\left(X_{1}\right)\leq E_{\alpha }\left(X_{2}\right).$*
**(ii)**  *If $X_{1}\geq_{mrl}X_{2}$ and either $X_{1}$ or $X_{2}$ is DMRL, then $E_{\alpha }\left(X_{1}\right)\geq E_{\alpha }\left(X_{2}\right).$*

**Proof.**  We assume that the SF of $X_{i,\alpha },i=1,2,$ is given by $S_{i,\alpha }\left(x\right)=B_{\alpha }\left(S_{i}\left(x\right)\right),x>0.$ Let $X_{2}$ be IMRL. From (22), we obtain

$$\begin{array}{c} E_{\alpha }\left(X_{1}\right)=E\left[m_{1}\left(X_{1,\alpha }\right)\right]\leq E\left[m_{2}\left(X_{1,\alpha }\right)\right]\leq E\left[m_{2}\left(X_{2,\alpha }\right)\right]=E_{\alpha }\left(X_{2}\right). \end{array}$$

The first inequality is due to $X_{1}\leq_{mrl}X_{2}$ and the last inequality follows since $X_{1}\leq_{st}X_{2}$ implies $X_{1,\alpha }\leq_{st}X_{2,\alpha }$ for α≥0 due to Lemma 1 and this is equivalent to $E\left[\psi \left(X_{1,\alpha }\right)\right]\leq E\left[\psi \left(X_{2,\alpha }\right)\right]$ for all functions ψ(·) with increasing behaviour. Suppose $X_{1}$ is IMRL. Then,

$$\begin{array}{c} E_{\alpha }\left(X_{1}\right)=E\left[m_{1}\left(X_{1,\alpha }\right)\right]\leq E\left[m_{1}\left(X_{2,\alpha }\right)\right]\leq E\left[m_{2}\left(X_{2,\alpha }\right)\right]=E_{\alpha }\left(X_{2}\right), \end{array}$$

and hence the result stated in (i) is obtained. The proof for assertion (ii) is quite similar. □

Hereafter, we show that the FGCRE is connected with the excess wealth order as another concept of variability. The excess wealth transform function has some links with the MRL function as

(27) $$m_{X}\left(F^{-1}\left(p\right)\right)=\frac{W_{X}\left(p\right)}{\bar{p}},\;p\in \left(0,1\right),\;\;\bar{p}=1-p.$$

Recently, Toomaj and Di Crescenzo [11] have shown that a similar result also holds for the GCRE. The FGCRE can be calculated from the excess wealth transform employing (22).

**Theorem** **13.** *For a non-negative RV X, we have, for all α≥0,*

(28) $$E_{\alpha }\left(X\right)=c\left(\alpha \right)\int_{0}^{1}m_{X}\left(F^{-1}\left(p\right)\right){\left[-\log\left(1-p\right)\right]}^{\alpha -1}\;dp.$$

It has been established by Fernández-Ponce et al. [19] that the variance of *X* can be measured by excess wealth as

$$\sigma^{2}\left(X\right)=\int_{0}^{1}{\left[m_{X}\left(F^{-1}\left(p\right)\right)\right]}^{2}\;dp.$$

Notice that $X_{1}\leq_{ew}X_{2}$ implies $\sigma^{2}\left(X_{1}\right)\leq \sigma^{2}\left(X_{2}\right)$ (cf. [12]). From (28), the following result is reached.

**Theorem** **14.** *If $X_{1}\leq_{ew}X_{2},$ then $E_{\alpha }\left(X_{1}\right)\leq E_{\alpha }\left(X_{2}\right),$ for any α≥0.*

Consequently,

$$X\leq_{disp}Y\;⟹\;X\leq_{ew}Y\;⟹\;E_{\alpha }\left(X\right)\leq E_{\alpha }\left(Y\right),$$

for any α≥0.

## 4. Dynamic FGRCE

The study of the times for events or the age of units is of interest in many fields. The FGCRE of $X_{t}$ is

(29) $$E_{\alpha }\left(t\right)=E_{\alpha }\left(X;t\right)=c\left(\alpha \right)\int_{t}^{\infty }\frac{S(x)}{S(t)}{\left[\Omega (x)-\Omega (t)\right]}^{\alpha }dx,\;t>0,$$

for all α≥0. It is clear that $E_{0}\left(t\right)=m\left(t\right)$. The HR of $X_{t}$ is λ(x+t) for x≥0. Hence, if *X* is IFR(DFR), then $X_{t}$ is also IFR(DFR) and, therefore,

(30) $$E_{\alpha_{1}}\left(X;t\right)\leq \left(\geq \right)E_{\alpha_{2}}\left(X;t\right),$$

for all $0\leq \alpha_{1}\leq \alpha_{2}$. On the other hand, by using the generalized binomial expansion, for all α≥0,

Eα(X;t)=c(α)∫t∞S(x)S(t)Ω(x)−Ω(t)αdx=c(α)S(t)∑k=0∞αk(−1)kΩ(t)k∫t∞S(x)Ω(x)α−kdx.

In analogy with Theorem 1, the next result is procured:

(31) $$\begin{array}{c} E_{\alpha }\left(X;t\right)=E\left[X_{\alpha +1}-X_{\alpha }\;|\;X>t\right]=c\left(\alpha \right)\int_{0}^{\infty }S_{t}\left(x\right){\left[\Omega_{t}\left(x\right)\right]}^{\alpha }\;dx,\;\alpha \geq 0. \end{array}$$

The dynamic version of identity (22) follows from the following identity,

(32) $$f_{\alpha }\left(x\;|\;t\right):=\frac{{\left[\Omega \left(x\right)-\Omega \left(t\right)\right]}^{\alpha -1}}{\Gamma (\alpha )}\frac{f(x)}{S(t)},\;x\in \left[t,+\infty \right),t\geq 0,$$

which is the PDF of the conditional RV $\left[X_{\alpha }\;|\;X>t\right]$, α>0. This is the generalization of expression given in (33) of Toomaj and Di Crescenzo [11] when α is a positive integer. The result in Theorem 10 of Toomaj and Di Crescenzo [11] is generalized as follows:

**Theorem** **15.** *In the setting of Theorem 7, for non-negative α and t,*

(33) $$E_{\alpha }\left(X;t\right)=E\left[m\left(X_{\alpha }\right)\;|\;X>t\right].$$

**Theorem** **16.** *For any t≥0 and for all α≥0, it holds that*

$$\frac{1}{\alpha }\;\mathrm{Cov}\left[X_{\alpha },\Omega \left(X_{\alpha }\right)\;|\;X>t\right]=E_{\alpha }\left(X;t\right).$$

**Proof.**  Let us denote $A_{t}=\left[X>t\right]$. We obtain

$$\mathrm{Cov}\left[X_{\alpha },\Omega \left(X_{\alpha }\right)\;|\;A_{t}\right]=E\left[X_{\alpha }\;\Omega \left(X_{\alpha }\right)|A_{t}\right]-E\left[X_{\alpha }|A_{t}\right]\;E\left[\Omega \left(X_{\alpha }\right)|A_{t}\right].$$

From (32), one can easily obtain

$$E\left[X_{\alpha }\;\Omega \left(X_{\alpha }\right)|A_{t}\right]=\int_{t}^{\infty }x\;\Omega \left(x\right)\;f_{\alpha }\left(x\;|\;t\right)\;dx=\alpha \;E\left[X_{\alpha +1}\;|\;A_{t}\right]+\Omega \left(t\right)\;E\left[X_{\alpha }\;|\;A_{t}\right]$$

and

$$E\left[\Omega \left(X_{\alpha }\right)\;|\;A_{t}\right]=\int_{t}^{\infty }\Omega \left(x\right)\;f_{\alpha }\left(x\;|\;t\right)\;dx=\alpha +\Omega \left(t\right),$$

so that

$$\mathrm{Cov}\left[X_{\alpha },\Omega \left(X_{\alpha }\right)\;|\;A_{t}\right]=\alpha \;\left(E\left[X_{\alpha +1}\;|\;A_{t}\right]-E\left[X_{n}\;|\;A_{t}\right]\right).$$

The result now follows from (31). □

For t=0, Theorem 16 is reduced to the next achievement:

**Corollary** **1.** *For all α≥0,*

$$\frac{1}{\alpha }\;\mathrm{Cov}\left(X_{\alpha },\Omega \left(X_{\alpha }\right)\right)=E_{\alpha }\left(X\right).$$

In a similar manner as in Theorem 9, the following bounds for the dynamic measure (4) are derived for t>0:

(34) $$E_{\alpha }\left(X;t\right)\left\{\begin{array}{c} \leq \frac{c\left(\alpha \right){\left[E\left(X;t\right)\right]}^{\alpha }}{{\left[m\left(t\right)\right]}^{\alpha -1}},\;\mathrm{if}\;0\leq \alpha \leq 1\\ \geq \frac{c\left(\alpha \right){\left[E\left(X;t\right)\right]}^{\alpha }}{{\left[m\left(t\right)\right]}^{\alpha -1}},\;\mathrm{if}\;\alpha \geq 1 \end{array}.\right.$$

The following theorem with the same arguments as in the proof of Theorem 10 gives the dynamic version of the FGCRE.

**Theorem** **17.** *For X with a finite MRL function and finite* E(X;t),*for all* α≥0,*we have:*

**(i)**  *$E_{\alpha }\left(X;t\right)\geq C_{\alpha }e^{H(X;t)}$ in which $C_{\alpha }$ is as before. H(X;t) denotes the dynamic Shannon entropy introduced in [20].*
**(ii)** 
$E_{\alpha }\left(X;t\right)\geq c\left(\alpha \right)\int_{t}^{\infty }{\left(1-\frac{F(x)}{F(t)}\right)}^{\alpha }\frac{S(x)}{S(t)}dx.$

Moreover, following the proof of Theorem 11, a couple of upper bounds for the dynamic FGCRE are acquired. The definition and properties of the variance residual lifetime (VRL) function in the context of lifetime data analysis have been studied in Gupta [21], Gupta et al. [22] and Gupta and Kirmani [23], among others.

**Theorem** **18.** *Let X have a VRL function* $\sigma^{2}\left(X;t\right)$*and finite dynamic FGCRE* $E_{\alpha }\left(X;t\right),$*for all* α≥0.*Then,*

**(i)**  *$E_{\alpha }\left(X;t\right)\leq \frac{\sqrt{\Gamma (2\alpha -1)}}{\Gamma (\alpha )}\sigma \left(X;t\right),$ for all α≥0.5.*
**(ii)**  *$E_{\alpha }\left(X;t\right)\leq {\left(\frac{\alpha e^{-1}}{1-\beta }\right)}^{\alpha }c\left(\alpha \right)\pi_{\beta }\left(X;t\right)$, where β=α for 0≤α≤1 and β=1/α for α≥1 and $\pi_{\beta }\left(X;t\right)=\int_{t}^{\infty }{\left(\frac{S(x)}{S(t)}\right)}^{\alpha }dx,\;t>0.$*

Now, we give an expression for the derivative of $E_{\alpha }\left(X;t\right).$

**Theorem** **19.** *We have*

(35) $$E_{\alpha }^{\prime }\left(X;t\right)=\lambda \left(t\right)\left[E_{\alpha }\left(X;t\right)-E_{\alpha -1}\left(X;t\right)\right],$$

*for all α≥1.*

**Proof.**  The relation (33) gives

$$E_{\alpha }\left(X;t\right)S\left(t\right)=\int_{t}^{\infty }\frac{{\left[\Omega \left(x\right)-\Omega \left(t\right)\right]}^{\alpha -1}}{\Gamma (\alpha )}f\left(x\right)\;m\left(x\right)\;dx.$$

By differentiating, we obtain

$$E_{\alpha }^{\prime }\left(X;t\right)S\left(t\right)-f\left(t\right)E_{\alpha }\left(X;t\right)=-\frac{\lambda (t)(\alpha -1)\Gamma (\alpha -1)}{\Gamma (\alpha )}\int_{t}^{\infty }\frac{{\left[\Omega \left(x\right)-\Omega \left(t\right)\right]}^{\alpha -2}}{\Gamma (\alpha -1)}f\left(x\right)\;m\left(x\right)\;dx.$$

Applying Γ(α)=(α−1)Γ(α−1) and using again (33),

$$E_{\alpha }^{\prime }\left(X;t\right)S\left(t\right)-f\left(t\right)E_{\alpha }\left(X;t\right)=-\lambda \left(t\right)E_{\alpha }\left(X;t\right),$$

that is, (35) holds. □

The preceding theorem can be applied to present the following theorem:

**Theorem** **20.** *If X is IFR (DFR), then $E_{\alpha }\left(X;t\right)$ is decreasing (increasing) for all α≥1.*

**Proof.**  The result is immediate for α=1 since $E_{\alpha }\left(X;t\right)=m\left(t\right)$ and since the IFR (DFR) property is stronger than the DMRL (IMRL) property. For all α>1, using relation (30), we have

$$E_{\alpha }\left(X;t\right)\geq \left(\leq \right)E_{\alpha -1}\left(X;t\right),$$

which validates the theorem by using Theorem 19. □

Let us define a new aging notion based on the FGCRE.

**Definition** **1.** *The RV X has an increasing (decreasing) dynamic FGCRE of order α, and denote it by $IDFE_{\alpha }\left(DDFE_{\alpha }\right)$ if $E_{\alpha }\left(X;t\right)$ is increasing (decreasing) in t.*

We note that the $IDFE_{0}$ and $DDFE_{0}$ classes correspond to the IMRL (increasing MRL) and DMRL (decreasing MRL) classes, respectively. In the next theorem, we prove $IDFE_{\alpha -1}\left(DDFE_{\alpha -1}\right)$ is a subclass of $IDFE_{\alpha }\left(DDFE_{\alpha }\right)$ for all α≥1.

**Lemma** **2.** *Let $E_{\alpha }\left(X;0\right)<\infty$ for a fixed α≥1. Then*

(36) $$E_{\alpha }\left(X;t\right)=\frac{1}{S(t)}\int_{t}^{\infty }E_{\alpha -1}\left(X;x\right)\;f\left(x\right)\;dx.$$

Under the assumptions of Lemma 2, $E_{\alpha }\left(X;t\right)$ is an absolutely continuous function. Furthermore, for α=0, then $m\left(t\right)=E_{0}\left(X;t\right)$ is also absolutely continuous under the hypothesis that μ<∞. Moreover, we have the following result.

**Theorem** **21.** *If X is $IDFE_{\alpha -1}\left(IDFE_{\alpha -1}\right),$ then X is $IDFE_{\alpha }\left(IDFE_{\alpha }\right)$ for all α≥1.*

**Proof.**  Suppose that *X* is $IDFE_{\alpha -1}$. Then, by using (36), we obtain

$$\begin{array}{ccc} E_{\alpha }\left(X;t\right) & = & \frac{1}{S(t)}\int_{t}^{\infty }E_{\alpha -1}\left(X;x\right)\;f\left(x\right)\;dx\\ & = & \frac{1}{S(t)}\int_{t}^{\infty }E_{\alpha -1}\left(X;t\right)\;f\left(x\right)\;dx\\ & = & E_{\alpha -1}\left(X;t\right), \end{array}$$

for all t≥00. Then (35) yields $E_{\alpha }\left(X;t\right)$ and *X* is $IDFE_{\alpha }$. The proof is similarly carried out when *X* is $DDFE_{\alpha }$. □

From Theorem 21, we can conclude that

$$IDFE_{\alpha_{1}}⟹IDFE_{\alpha_{2}}$$

and

$$DDFE_{\alpha_{1}}⟹DDFE_{\alpha_{2}}$$

for all $1\leq \alpha_{1}\leq \alpha_{2}.$ An immediate consequence of the above relation is that

$$IMRL⟹IDFE_{\alpha }\;\mathrm{and}\;DMRL⟹DDFE_{\alpha }$$

for all α≥0. We remark that Navarro et al. (2010) provided some examples showing that an RV *X* is $IDGCRE_{1}\left(DDGCRE_{1}\right)$ but it is not IMRL (DMRL). However, Navarro and Psarrakos [24] by some counterexamples showed that *X* is neither IMRL (DMRL) nor $IDGCRE_{1}\left(DDGCRE_{1}\right),$ but it is included in the class $IDGCRE_{\alpha }\left(DDGCRE_{\alpha }\right)$ when α is an integer value. Hence, the result holds for all α≥1.

This section is closed by introducing the dynamic normalized version of the FGCRE as follows:

(37) $${\mathrm{NE}}_{\alpha }\left(X;t\right)=\frac{E_{\alpha }\left(X;t\right)}{m(t)},$$

for all t>0.

**Theorem** **22.** *Let X have a finite normalized FGCRE ${\mathrm{NE}}_{\alpha }\left(X;t\right).$ If X is IMRL (DMRL), then ${\mathrm{NE}}_{\alpha }\left(X;t\right)\geq \left(\leq \right)1$ for all t>0.*

**Proof.**  Since *X* is IMRL (DMRL) based on the assumption, we have

$$\frac{m(x)}{m(t)}\geq \left(\leq \right)1,\;\forall x\geq t.$$

Therefore, from Equations (33) and (37), we obtain

$${\mathrm{NE}}_{\alpha }\left(X;t\right)=\int_{t}^{\infty }\left[\frac{m(x)}{m(t)}\right]f_{\alpha }\left(x\;|\;t\right)dx\geq \left(\leq \right)\int_{t}^{\infty }f_{\alpha }\left(x\;|\;t\right)dx=1,$$

from which we have the result. □

**Figure 3:**
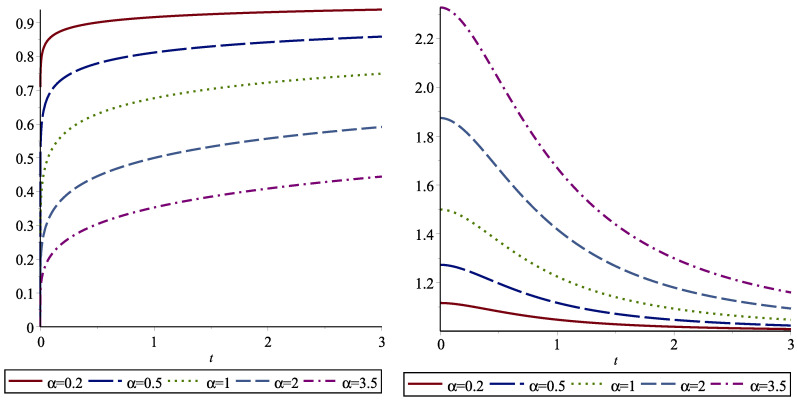
The dynamic normalized FGCRE for the Weibull distribution given in case (ii) of Table 4, with k=0.2 (**left** panel) and k=2 (**right** panel) as a function of *t* for various values of α=0.2,0.5,1,2,3.5.

In Table 4, we give the dynamically normalized FGCREs for some distributions. For example, we present the dynamically normalized FGCRE of the Weibull distribution in Figure 3. We note that *X* is IMRL when k≥1 and *X* is DMRL when 0≤k≤1.

Eventually, the inequalities given in (25) can be developed as

$${\mathrm{NE}}_{\alpha }\left(X;t\right)\left\{\begin{array}{c} \leq c\left(\alpha \right){\left[\mathrm{NE}\left(X;t\right)\right]}^{\alpha },\;\mathrm{if}\;0\leq \alpha \leq 1\\ \geq c\left(\alpha \right){\left[\mathrm{NE}\left(X;t\right)\right]}^{\alpha },\;\mathrm{if}\;\alpha \geq 1 \end{array}.\right.$$

The inequalities given above are very useful when the dynamic FGCRE has a complicated form.

## Figures and Tables

**Table 1:** FGCREs for a number of distributions.

Distribution	S(x)	$E_{\alpha }\left(X\right)$
Uniform(0,b)	$1-\frac{x}{b},\;0\leq x\leq b$	$\frac{b}{2^{\alpha +1}},\;b>0.$
Weibull(1,k)	$e^{-x^{k}},\;x>0$	$\frac{c(\alpha +1)}{kc(\alpha +\frac{1}{k})},\;k>0.$
Burr Type II(c,k)	${\left(1+x^{c}\right)}^{-k},\;x>0$	kαc∑i=0∞1c−1i(−1)i(k+i−1c)α+1,c,k>0.
Beta(1,b)	${\left(1-x\right)}^{b},\;0\leq x\leq 1$	$\frac{b^{\alpha }}{{\left(b-1\right)}^{\alpha +1}},\;b>1.$

**Table 2:** FGCREs for several distributions.

Distribution	S(x)	${\mathrm{NE}}_{\alpha }\left(X\right)$
Uniform	$1-\frac{x}{b},\;0\leq x\leq b$	$\frac{1}{2^{\alpha }},\;b>0.$
Weibull	$e^{-x^{k}},\;x>0$	$\frac{c\left(\alpha \right)c\left(1+\frac{1}{k}\right)}{kc(\alpha +\frac{1}{k})},\;k>0.$
Burr Type II	${\left(1+x^{c}\right)}^{-k},\;x>0$	kα−1cB(k−1c,1+1c)∑i=0∞1c−1i(−1)i(k+i−1c)α+1,c,k>0. *
Beta	${\left(1-x\right)}^{b},\;0\leq x\leq 1$	$\frac{b^{\alpha }\left(b+1\right)}{{\left(b-1\right)}^{\alpha +1}},\;b>1.$

* *B*(·, ·) denotes the complete beta function.

**Table 3:** Numerical values of $E_{\alpha }\left(X_{1}\right)$ and $E_{\alpha }\left(X_{2}\right)$ described in Example 3.

$k_{1}$	$k_{2}$	α	$E_{\alpha }\left(X_{1}\right)$	$E_{\alpha }\left(X_{2}\right)$	$k_{1}$	$k_{2}$	α	$E_{\alpha }\left(X_{1}\right)$	$E_{\alpha }\left(X_{2}\right)$
0.2	0.5	0.5	0.2329	0.2271	2	3	0.5	0.1570	0.1297
		1.0	0.1843	0.1574			1.0	0.0876	0.0681
		1.5	0.1503	0.1155			1.5	0.0551	0.0413
		2.0	0.1221	0.0858			2.0	0.0364	0.0266
		2.5	0.0980	0.0638			2.5	0.0247	0.0176

**Table 4:** FGCREs, MRLs and normalized FGCREs for some distributions.

Distribution	S(x)	Parameters	$E_{\alpha }\left(X;t\right)$	m(t)	${\mathrm{NE}}_{\alpha }\left(X;t\right)$
Uniform(a,b)	$\frac{b-t}{b-a},\;0\leq x\leq b$	0≤a<b	$\frac{\left(b-t\right)}{2^{\alpha +1}}$	$\frac{\left(b-t\right)}{2}$	$2^{-\alpha }$
Weibull(c,k)	$e^{-cx^{k}},\;x>0$	c>0,k>0	$\frac{\Gamma_{1-1/k}\left(\alpha +1,ct^{k}\right)}{k\sqrt[{k}]{c}\Gamma \left(\alpha +1\right)}$	$\frac{\Gamma_{1-1/k}\left(1,ct^{k}\right)}{k\sqrt[{k}]{c}}$	$\frac{\Gamma_{1-1/k}\left(\alpha +1,ct^{k}\right)}{\Gamma \left(\alpha +1\right)\Gamma_{1-1/k}\left(1,ct^{k}\right)}$ *
Power(a,b,c)	${\left(\frac{b-t}{b-a}\right)}^{c},\;a\leq t\leq b$	0≤a<b,c>0	$\frac{c^{\alpha }\left(b-t\right)}{{\left(c+1\right)}^{\alpha +1}}$	$\frac{\left(b-t\right)}{\left(c+1\right)}$	${\left(\frac{c}{c+1}\right)}^{\alpha }$
Pareto(a,b)	${\left(\frac{a}{a+t}\right)}^{b},\;x\geq 0$	a>0,b>1	$\frac{b^{\alpha }\left(a+t\right)}{{\left(b-1\right)}^{\alpha +1}}$	$\frac{\left(a+t\right)}{\left(b-1\right)}$	${\left(\frac{b}{b-1}\right)}^{\alpha }$

* $\Gamma_{r}(m,t)=\int_{0}^{\infty }x^{m-1}{\left(x+t\right)}^{-r}e^{-x}dx$ denotes the generalized gamma function.

## Data Availability

No new data were created or analyzed in this study. Data sharing is not applicable to this article.
